# Conformal smoothing of mid-spatial frequency surface error for nano-accuracy Continuous Phase Plates (CPP)

**DOI:** 10.1038/s41598-020-59484-4

**Published:** 2020-02-13

**Authors:** Ci Song, Wanli Zhang, Feng Shi, Zhifan Lin, Xuqing Nie

**Affiliations:** 1 College of Intelligent Science and Technology, National University of Defense Technology, Changsha, 410073 China; 2 Hunan Key Laboratory of Ultra-Precision Machining Technology, Changsha, 410073 China; 3 Laboratory of Science and Technology on Integrated Logistics Support, National University of Defense Technology, Changsha, 410073 China

**Keywords:** Mechanical engineering, Mechanical engineering, Optical materials and structures, Optical materials and structures

## Abstract

This paper presented a conformal smoothing theory, and smoothing capability evaluation was established on the proposed theory. According to pressure distribution model, processing parameters have been optimized and the CPP sample with a size of 340 × 340 mm was applied in conformal smoothing. The middle spatial frequency was effectively corrected with the total polishing time of 750 min, and energy was constringed 32.2 times (improved from 57.68 nm^2^·mm to 1.79 nm^2^·mm). Meanwhile, surface roughness RMS (root mean square) maintained at the same scale (changed from 265.4 nm to 265.2 nm). Parametric conformal smoothing was proven to be an effective method to control the middle spatial frequency error of CPPs.

## Introduction

Continuous phase plate (CPP) with continuously varying topographical microstructures is a typical diffractive optical element, and it has wide spread applications in modern optics field of beam shaping, compensation and modulation^1,2^ CPPs surface have spatial microstructure, fractal surface, and large grads, which are different from traditional optics and common microstructure array component. The phase unit characteristic size, PV (peak-to-valley), and surface grads of typical CPPs are 5~50 mm, 1~10 μm, and 1~5 μm/cm respectively. The requirements on gradient and spatial frequency make it difficult to fabricate.

Magnetorheological finishing (MRF), an advanced optical finishing process combining interferometry, precision equipment, and computer control, has been an effective method to process CPPs^3–6^. During the polishing process, dwell time is accurately controlled to get CPPs structure with nanometer accuracy. However, MRF has a few matters lead to mid-to-high spatial frequency error:^7–10^ (1) removal function fluctuation including removal function size or removal efficiency fluctuation. (2) CCOS (computer controlled optical surfacing) convolutional residual error produced by manufacturing route. The mid-to-high spatial frequency error on CPPs surface leads to serious problems such as grating effect and far-field light intensity modulation.

Limited by the size of polishing tool in MRF, IBE and atmospheric pressure plasma processing (APPP) have been chosen to fabricate CPPs^11,12^. For IBE, fabrication route and removal function fluctuation restrict the improvement of surface precision, while post-treatment need be applied to remove residues in APPP.

Existing smoothing theories and technologies have effectively controlled mid-to-high spatial frequency error. Researchers have manufactured many aspheric surfaces using flexible plates^13–16^. Mehta Firstly derived pressure distribution model of flexible smoothing plate in aspheric surface manufacture based on Bridge Model. Dae Wook Kim developed the smoothing model of RC plate and then compared smoothing factors of pitch tools and RC plates^17^. Walker proposed pseudo-random tool paths for CNC sub-aperture polishing^18^. Yifan Dai proposed local random processing path based on maximum entropy method^19^. Hon-Yuen Tam proposed Peano-like paths for sub-aperture polishing of optical aspherical surfaces^20^. All of the techniques were confirmed that had good smoothing effects, while they could not be used to fabricate CPPs because of the change in microstructure.

Most of existing smoothing theories concentrate on a single frequency, while the relationships of low-spatial frequency surface and middle spatial frequency could not be described simultaneously. Hence, conformal smoothing should be developed, factors to quantitatively evaluate smoothing and conformal capability should be completed. Conformal smoothing aims to smooth mid-to-high spatial frequency of millimeter level and keep specific low-spatial frequency surface precision. Conformal SP should be optimized.

This paper focuses on theoretic analysis of SP, optimization of conformal smoothing tool, and engineering applications of conformal smoothing. Firstly, the conception of smoothing ratio was proposed to characterize the effect of smoothing and conformability based on pressure distribution model. Then, the smoothing tool was optimized by theoretically analyzing, simulating, and experimentally verifying. Finally, the optimized tool was applied in the smoothing of 340 mm × 340 mm CPP, where mid-to-high spatial frequency errors were effectively controlled.

## Theory of Conformal Smoothing

### Pressure distribution model

To calculate the pressure distribution, a generalized model is established in this research. In this model, the contact between polishing pad and component is considered to be elastic and the component is assumed to be rigid. Effects friction and others are ignored, in order to simplify the model^21,22^.

**Figure 1:**
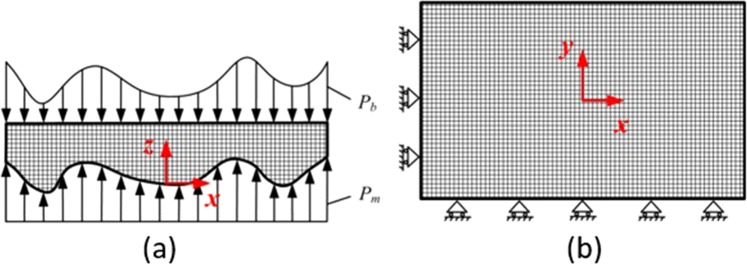
Meshed Finite Element Method model of the polishing pad: (**a**) Front view (**b**) top view.

As shown in Fig. 1, P_b_ is the force exerted by polishing pad. P_m_ is the supporting force.

According to laws of minimum potential energy and Hooke’s, the governing matrix equation of hexahedral elements separated from pressure distribution model could be defined as follows:

1 $$\frac{\partial {G}_{0}}{\partial {C}_{0}}={F}_{0}={K}_{0}{C}_{0},\,{K}_{0}={\int }_{v}{B}_{0}^{T}{D}_{0}{B}_{0}dv$$

F_0_ is the nodal force vector. K_0_ is the element stiffness matrix. C_0_ is the nodal displacement vector. B_0_ is the strain-displacement matrix. D_0_ is the elastic stress-strain relation matrix.

Both matrices mentioned in last equation are related to properties of polishing tool and mid-spatial frequency error (MSFR)^23^.

Then, integrated every vector in Eq. (1) and the whole matrix could be defined as follows:

2 $${F}_{all}={K}_{all}\cdot {C}_{all}$$

Meanwhile, the boundary conditions could be described as following equations set before solving the model.

3 $$\{\begin{array}{c}{C}_{m}={\rm{error}}\\ \frac{\partial ({C}_{{\rm{b}}})}{\partial z}=0\\ {u}_{x=0}=0,\,{v}_{y=0}=0\\ \sum {F}_{b}=-\sum {F}_{m},\,\sum {F}_{b}=f\cdot \frac{{S}_{u}}{S}\end{array}$$

C_m_ and C_b_ are the displacements of lower and upper surface. error is the MSFR on the mirror. u and v are the displacements in the x and y direction. F_b_ is the nodal force of upper surface. F_m_ is the nodal force of lower surface. *f* is the whole force applied on the lap. S_u_ is the upper surface area. S is the whole area of the pad.

In the model, the pressure distribution on upper and lower surface can be attained from F_b_ and F_m_. Further, the material removal rate of MSFR can be calculated by Preston’s equation^24^.

### Smoothing ratio *K*′

Smoothing ratio *K*′ represents the efficiency of PV convergence of different frequency errors. According to Preston’s equation it could be defined as follows:

4 $$K{\prime} =\frac{{\rm{d}}(p{v}_{f1})}{{\rm{d}}(p{v}_{f2})}=\frac{\varDelta {p}_{f1}}{\varDelta {p}_{f2}}$$

ΔP_f1_ is the pressure of error frequency f_1_. ΔP_f2_ is the pressure of error frequency f_2_.

Based on Eq. (4), the following conclusions can be obtained.

1. $K{\prime}$ can describe the smoothing effects of different spatial frequency surface errors with variant smoothing tool.
2. The larger the smoothing ratio $K{\prime}$ is, the better the smoothing ability of mid-to-high spatial frequency surface error, and the better the conformal ability of low-spatial frequency surface error will be.
3. Based on pressure distribution model, the smoothing ratio K’ would change along with parameters in smoothing process.

## Conformal Smoothing Validation

### Optimization of smoothing tool

There are a lot of parameters affecting the smoothing ratio including the periodicity of surface error *a*, the elastic modulus *E*, the Poisson’s ratio *v*, the thickness of base *h*, and the amplitude of surface error PV. The periodicity of surface error *a* and the amplitude of surface error PV are mainly dominated by the surface error, and the other parameters are dominated by the material and thickness of polishing tool. In the optimized process of smoothing tool, the material and thickness of base will be optimized to maximize the smoothing ratio in a reasonable range. In smoothing process, the size of smoothing tool is one half of low-spatial frequency error periodicity and the convergence in full bands of errors is achieved^25^.

**Figure 2:**
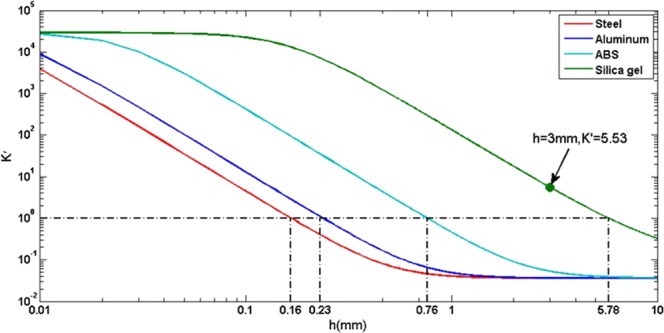
Simulation of the smoothing ratio $K{\prime}$ with different materials and thickness.

To optimize the material of base, four different materials (steel, aluminum, ABS resin, silica gel) are compared. The elastic modulus *E* and Poisson’s ratio *v* are both listed in Table 1. To understand the SP, the smoothing ratio of the 30 mm periodic surface error to 1 mm tool-path with different base thickness are simulated respectively, as shown in Figs. 1 and 2. In the simulation process, the compression rigidity of pitch *Kc* = 4.4e^9^N/m^3 ^^26^.

**Table 1:** Physical properties of four materials.

Material	E(Pa)	ν
Steel	2.09E + 11	0.269
Aluminum	6.90E + 10	0.33
ABS	2.00E + 09	0.39
Silica gel	1.00E + 05	0.35

In Fig. 2, we can find that the smoothing ratio $K{\prime}$ reduces rapidly when the thickness of base increases, and it tends to be the same when the base thickness further increase. Also, when the elastic modulus *E* decreases, the smoothing ratio increases rapidly. To obtain a large smoothing ratio, the soft base material is recommended. As limited by the structure of the smoothing tool, it is difficult to make the thickness of base less than 3 mm. Therefore, the material and thickness of base are chosen as Silica gel and 3 mm, respectively. The simulation results show that the smoothing ratio is 5.53 when 3 mm Silica gel is used as the smoothing tool.

### Smoothing experiments

**Figure 3:**
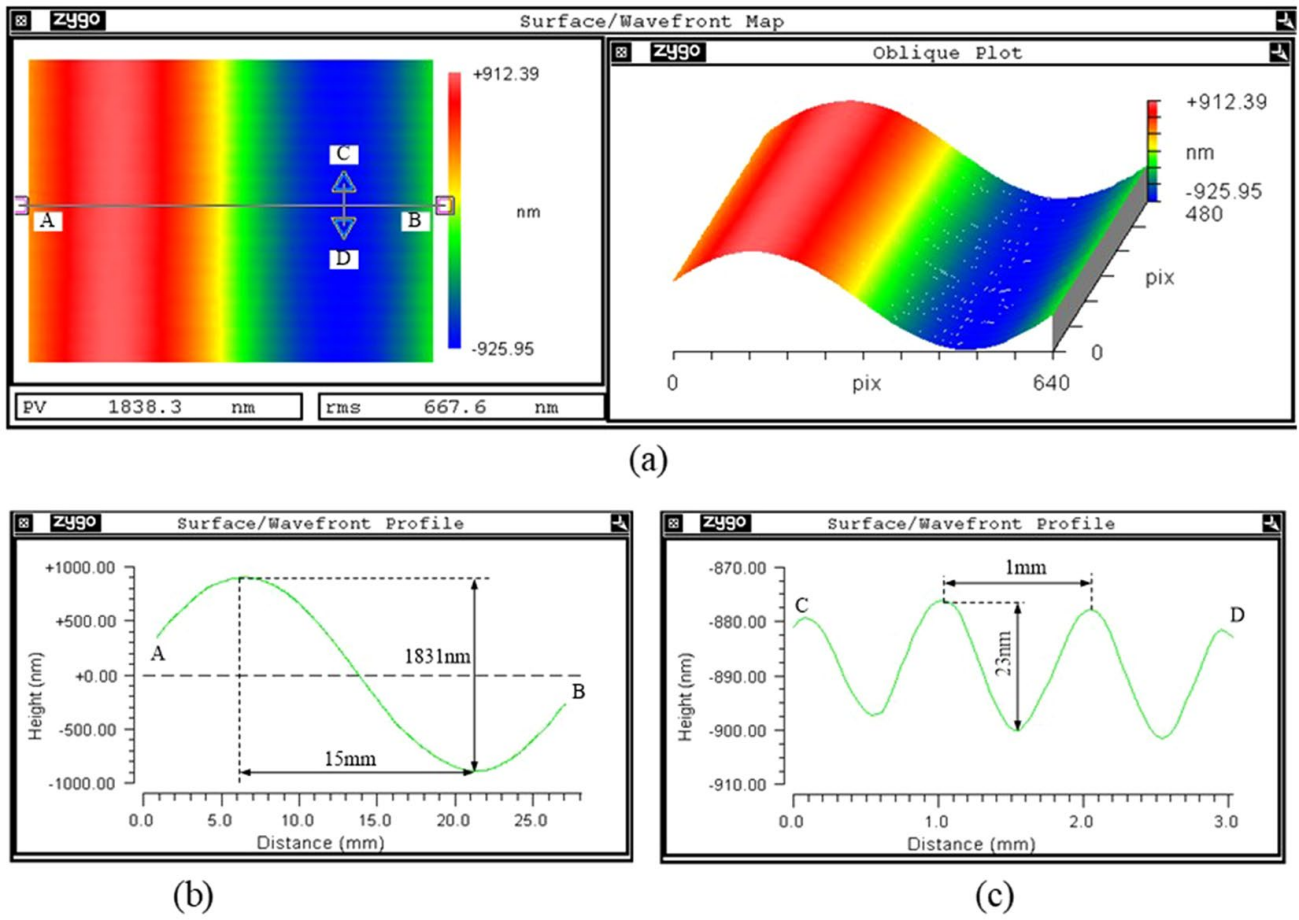
Sine surface error manufactured by MRF. (**a**) Local surface map distribution, (**b**) sectional diagram in the horizon direction (**c**) sectional diagram in the vertical direction.

To validate the conformal smoothing results, different spatial frequency surface error is used to smooth. As the typical spatial period and amplitude of surface error is 1~50 mm and 0.1~5 μm, we choose two types of sine spatial frequency surface error to perform the smoothing experiments. One type of sine surface error is 30 mm periodic and 1831 nm PV, and another type of sine surface error is 1 mm periodic and 23 nm PV. The two types of sine surface error which both manufactured by MRF polishing tool are on the same workpiece but with different direction (one is on x direction, another is on y direction), as shown in Fig. 3(a).

**Figure 4:**
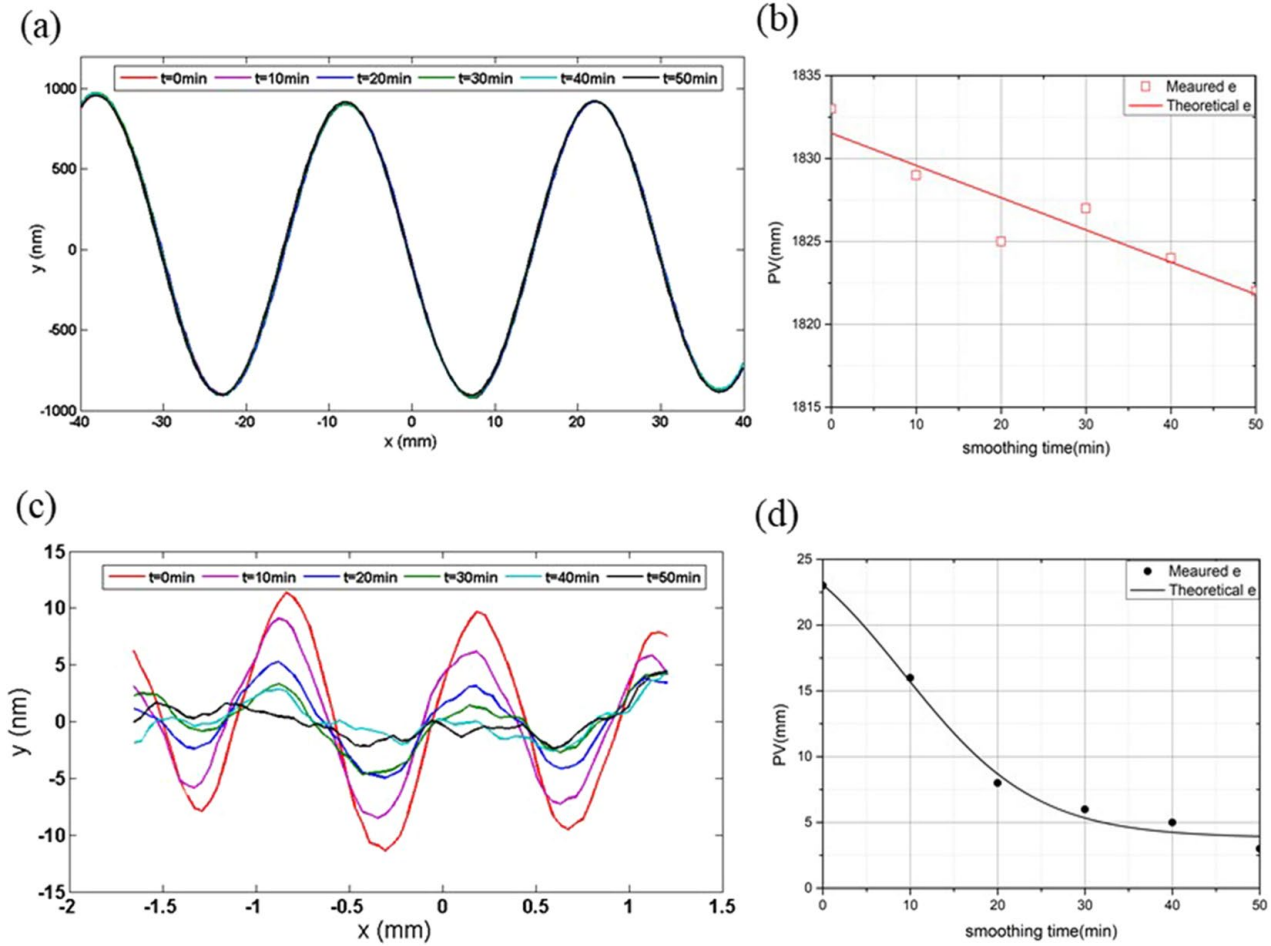
Convergence process with two different periodic surface errors. (**a**) Sectional diagram of 30 mm periodic surface error; (**b**) PV convergence curve of 30 mm periodic surface error; (**c**) sectional diagram of 1 mm periodic surface error; (**d**) PV convergence curve of 1 mm periodic surface error.

The smoothing parameters are all listed in Table 2. The time of one smoothing run is 10 min, and the PV of two types of surface error are measured and depicted in every smoothing run, as shown in Fig. 4

**Table 2:** Smoothing parameters.

Material of base	Silica gel
Thickness of base	3 mm
Thickness of pitch	2 mm
Diameter of tool	15 mm
Pressure of smoothing	0.05 MPa
Revolution speed of tool	100 rpm
Spin speed of tool	105 rpm
Smoothing tool-path	raster
Smoothing time in a run	10 min
Polishing abrasive	CeO_2_
Abrasive size	1 μm

**Figure 5:**
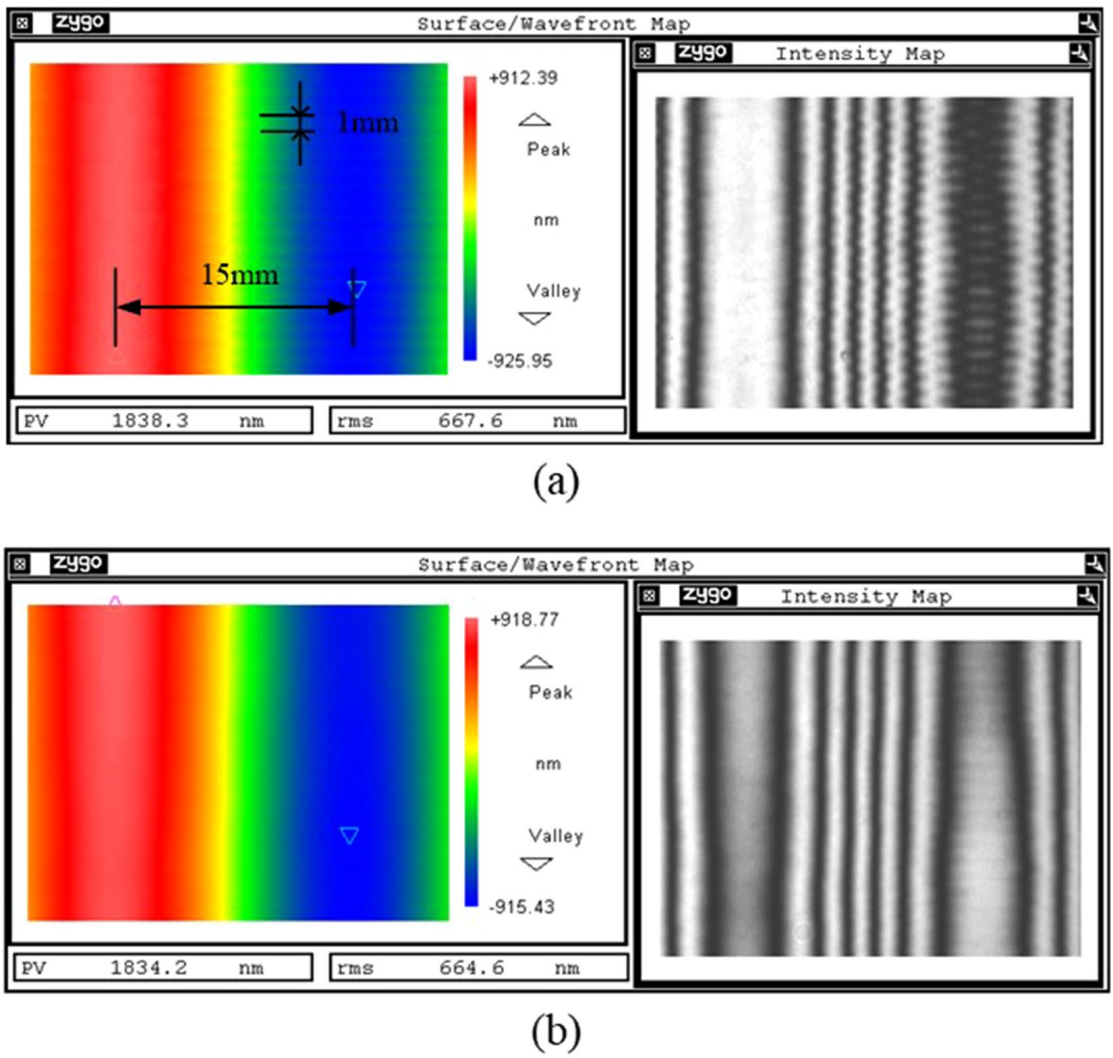
Smooth results (**a**) surface map distribution before the SP; (**b**) surface map distribution after 5 smoothing run.

Although the amplitude of 30 mm periodic surface error is higher than the amplitude of 1 mm periodic surface error, the PV convergence ratio of 1 mm periodic surface error is the faster one. It is observed that the 1 mm periodic surface error can be smooth in 5 smooth run process. However, the 30 mm periodic surface error almost the same in the 5 smooth run process and its PV value changes from 1831.5 nm to 1822.5 nm. The change of surface feature before and after the SP are shown in Fig. 5. In Fig. 5(a), not only the 30 mm periodic surface error but also the 1 mm periodic surface error are both obvious in the interferometry image. In Fig. 5(b), it is found that the 1 mm periodic surface error almost disappears, but the 30 mm periodic surface error still exists. This experiment demonstrates the smooth effectivity of mid-to-high spatial frequency surface error. It also demonstrates the conformal ability of low-spatial frequency surface error.

**Figure 6:**
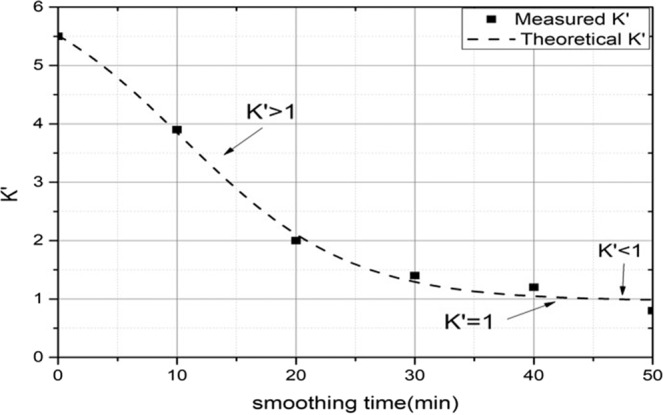
The smoothing ratio curve in the SP.

The smoothing ratio is also investigated in every smoothing run process, as shown in Fig. 6. The initial actual smoothing ratio is 5.53 which is almost agree with the theoretical value of 5.51. The smoothing ratio reduces with the increasing of smoothing times and runs. The final actual smoothing ratio is 0.80 which is also agreement with the theoretical value of 0.91.

Based on Fig. 6, the conformal SP can be divided into three steps. (1) At the beginning of SP, the smoothing ratio $K{\prime}  > 1$. The convergence rate of 1 mm periodic surface error is faster than the 30 mm periodic surface error. It means that the smoothing effect of mid-to-high spatial frequency surface error is faster than the low-spatial frequency surface error. The smoothing effect plays a major role in the mid-to-high spatial frequency surface error, and the conformal effect plays a major role in the low-spatial frequency surface error in the first step. (2)When the smoothing ratio $K{\prime} =1$, there is a balance between smoothing effect and conformal effect. At this theoretical point, the smoothing rate of mid-to-high spatial frequency surface error is theoretically the same as the low-spatial frequency surface error. (3) When the smoothing ratio $K{\prime}  < 1$, the smoothing rate of mid-to-high spatial frequency surface error is less than the low-spatial frequency surface error. The remove of low-spatial frequency surface error plays a major role, which the conformal ability will decrease and the surface accuracy tends to be worse in this process.

## Application of Conformal Smoothing for CPP

**Figure 7:**
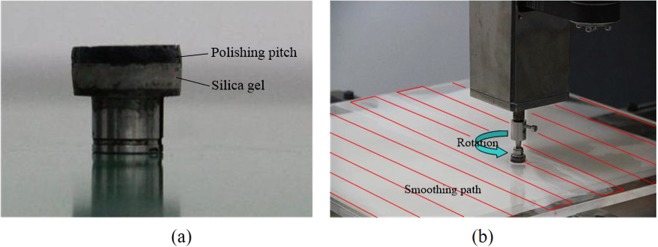
CPP SP. (**a**) Smoothing tool photo (**b**) smoothing tool-path photo.

A 340 mm × 340 mm fused silica CPP is used to perform the conformal SP, the smoothing tool and parameters are also the same with the above experiments, as listed in Table 2. The only difference is that the smoothing time of CCP increases to 150 min.The machine tool used to smooth is developed by ourselves. The smoothing tool mainly uses pitch and silica gel, as shown in Fig. 7(a). The smoothing tool-path is raster, as shown in Fig. 7(b), and there are 5 smoothing runs totally.

**Figure 8:**
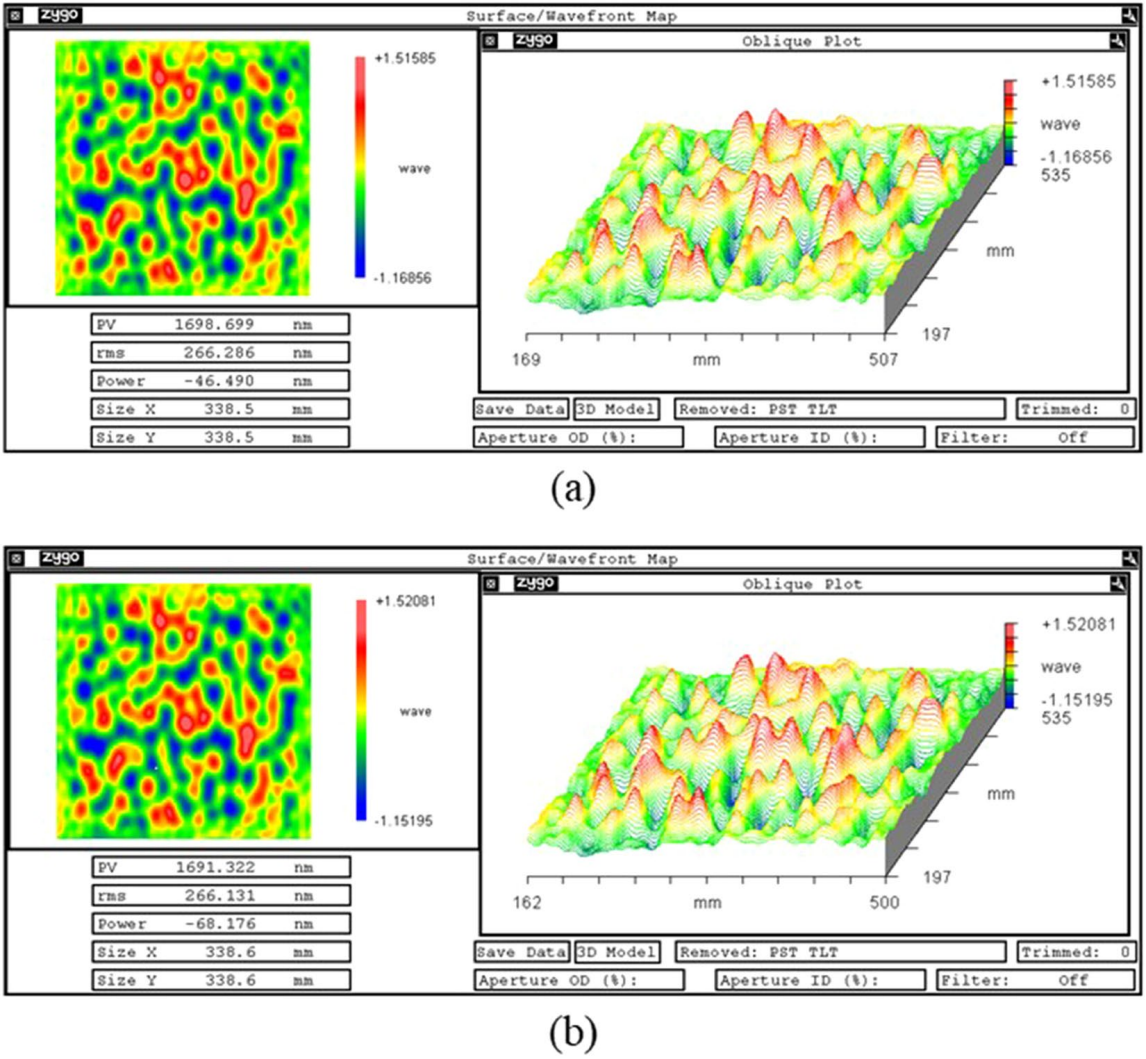
CCP smoothing results. (**a**) surface map before smoothing; (**b**) surface map after smoothing.

The full surface map before and after smoothing are both measured by Zygo interferometer VeriFire MST:GPI XP/D,a. Figure 8(a) shows the surface map before smoothing, the PV and RMS is 1698.699 nm and 266.286 nm, respectively. The PV and RMS is 1691.322 nm and 266.131 nm after 5 smoothing runs (750 minutes), as shown in Fig. 8(b).

The CCP is polished by MRF before, and the mesh size is 1 mm. Normally speaking, there will remain 1 mm periodic residual surface error on the surface. As the aperture of the interferometer is large, the data resolution is not enough to obtain the tool-path. To investigate the residual surface error before and after smoothing, the sub-aperture measurement is used. One sub-aperture can be zoomed largest, then the field range is 26.1 mm × 19.6 mm and the resolution is 0.0408 mm/pix. To compare the mid-to-high spatial frequency surface error before and after smoothing, the tested sub-aperture zone should be the same.

**Figure 9:**
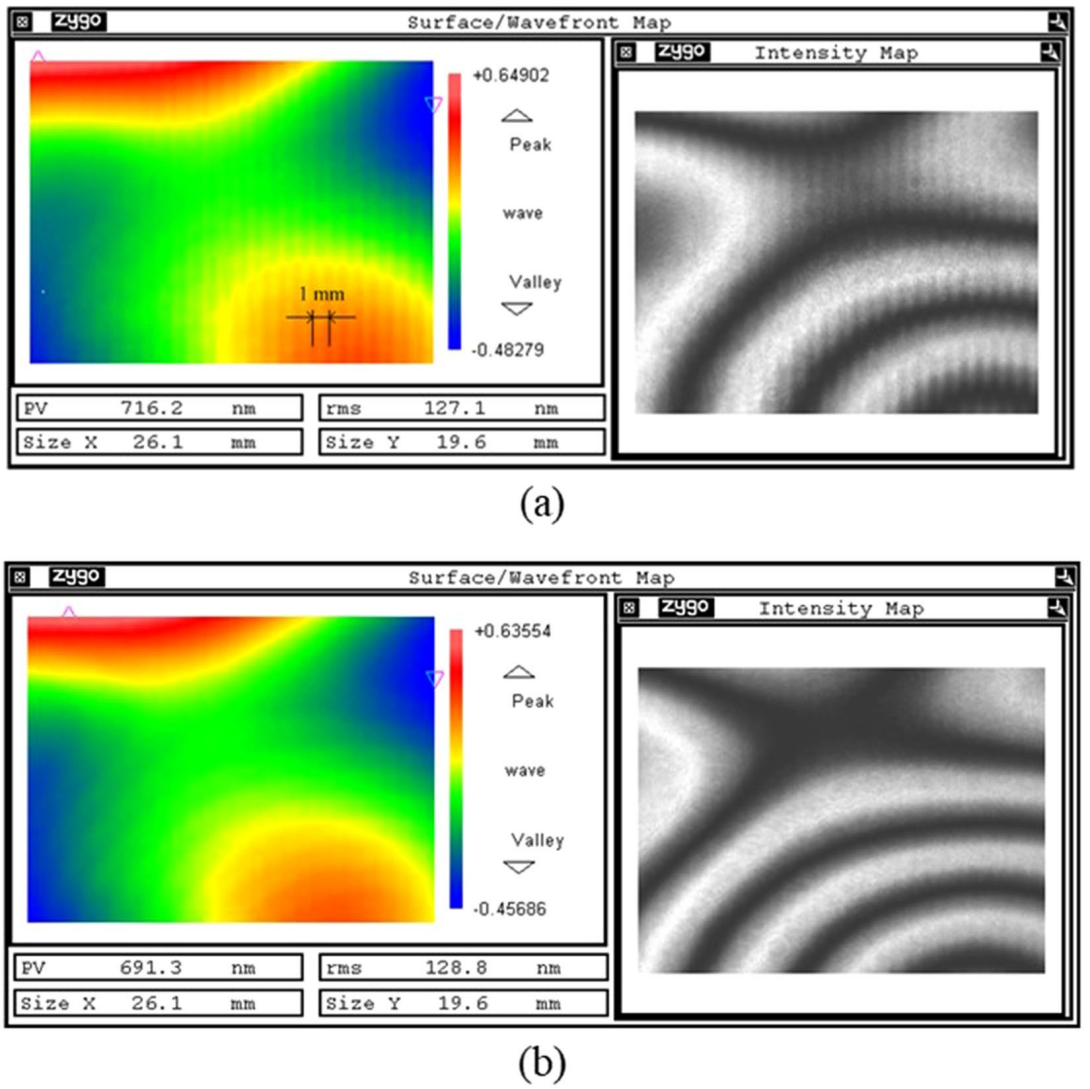
Sub-aperture test results. (**a**) Sub-aperture surface map before smoothing; (**b**) sub-aperture surface map after smoothing.

Figure 9 shows the same sub-aperture surface interferometry image before and after smoothing. There remain MRF tool-path residuals on the surface before smoothing. The max amplitude of tool-path residuals is almost 11.3 nm, and it reduces to 2.28 nm after smoothing. There are obvious ripples in the interferometry image before smoothing. However, after smoothing the ripples are difficult to observe on the surface. The results demonstrate that the SP can effectively remove the mid-to-high spatial frequency ripple generated in the MRF process.

**Figure 10:**
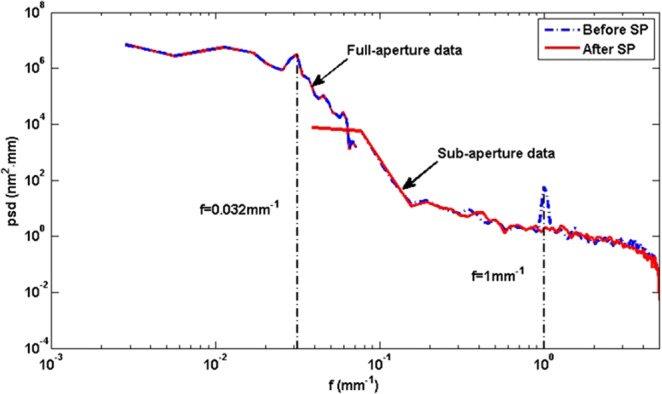
Full-aperture and sub-aperture PSD curve before and after SP.

To evaluate the SP further, the PSD curve before and after smoothing is calculated, as shown in Fig. 10. The full-aperture PSD mainly focuses on the low-spatial frequency surface error, in which the 30 mm periodic surface error feature (spatial frequency f = 0.032 mm^−1^) is almost the same. Besides, all the low-spatial frequency surface error are almost invariant, which demonstrate the conformal ability for low-spatial frequency surface error. The sub-aperture PSD mainly focuses on the mid-to-high spatial frequency surface error, in which the 1 mm periodic surface error feature (spatial frequency f = 1 mm^−1^) have been correctted obviously. The energy reduces from 57.68 nm^2^·mm to 1.79 nm^2^·mm, it demonstrates the smoothing ability for mid-to-high spatial frequency surface error.

## Conclusion

1. Based on the pressure distribution model, the smoothing ratio is proposed to describe the convergence rate of different periodic surface errors and evaluate the smoothing and conformal ability.
2. The smoothing tool is optimized based on the smoothing ratio. The simulation agrees with the smoothing experiments, which demonstrates the correctness of the smoothing ratio.
3. The optimized smoothing tool and parameters are used to smooth a 340 mm × 340 mm CPP sample polished by MRF. The PSD curve indicates that the low-spatial frequency surface error can be conformal and the tool-path can be eliminated effectively, the energy reduces from 57.68 nm^2^·mm to 1.79 nm^2^·mm and the surface accuracy RMS only changes from 266.3 nm to 266.1 nm in 5 smoothing run process with total 750 min. The CCP application demonstrates that the SP can not only conform the low-spatial surface accuracy but also eliminates the mid-to-high spatial frequency surface error.

## Data Availability

All data, models, during the study appear in the submitted article.
